# An Infant With Primrose Syndrome: A Case Report

**DOI:** 10.7759/cureus.46546

**Published:** 2023-10-05

**Authors:** Calista Long, Barry DeRose, Anthony B Lal, Elizabeth Imboden

**Affiliations:** 1 Pediatrics, Penn State College of Medicine, Hershey, USA; 2 Pediatrics, Drexel University College of Medicine, Philadelphia, USA; 3 Family Medicine, WellSpan Good Samaritan Hospital, Lebanon, USA; 4 Pediatrics, WellSpan York Hospital, York, USA

**Keywords:** rare genetic diseases, prenatal detection, prenatal genetic testing, corpus callosum agenesis, macrocephaly, autosomal dominant genetic disorder, intellectual disability (id), primrose syndrome

## Abstract

Primrose syndrome is a rare autosomal dominant disorder that is characterized by recognizable facial phenotype, sensorineural hearing loss, hypotonia, and developmental delay. All reported probands show de novo ZBTB20 pathogenic variant. Since its discovery in 1982, Primrose syndrome has remained an underdiagnosed condition. Awareness of presentation and prompt diagnostic workup are crucial for early identification and proper management. In this case report, we discuss a case of Primrose syndrome diagnosed in an infant born at Wellspan Hospital in York, PA. The patient exhibited classic phenotypic features, including a high hairline, high-arched palate, and brachycephaly at birth, as well as an absent corpus callosum observed on postnatal MRI and genotypic findings of a pathogenic variant in ZBTB20.

## Introduction

Primrose syndrome is a rare autosomal dominant disorder with less than 1000 reported cases in the United States [1]. It is characterized by intellectual disability, macrocephaly with developmental delay, altered glucose metabolism, sensorineural hearing loss, ocular anomalies, corpus callosum anomalies, cryptorchidism, calcification of the ear cartilage, and recognizable facial phenotype [2]. Facial characteristics include a prominent forehead, down-slanting palpebral fissures, ptosis, and large ears. Other findings, such as tall stature, hypothyroidism, and diabetes mellitus, have been reported, particularly in older patients [3]. The first recorded description of Primrose syndrome was made by David Primrose in 1982, describing a 33-year-old male with intellectual disability, muscle weakness of the lower limbs, calcified ear flaps, bone abnormalities, and a torus palatinus [3]. Primrose syndrome is an autosomal dominant disorder with all reported probands showing de novo ZBTB20 pathogenic variant [2]. ZBTB20 is located at chromosome position 3q13.31 and encodes a zinc finger protein containing transcription factors involved in glucose metabolism, postnatal growth, and neurogenesis [4]. In this case report, we present a case of Primrose syndrome diagnosed in utero via ultrasound, MRI, and genetic testing. The postnatal presentation was consistent with Primrose syndrome.

## Case presentation

Our patient, a 40-weeks and 1-day-old, appropriate for gestational age (AGA) female infant, was born via C-section delivery due to placental abruption to a G2P2002 mother. The prenatal infectious screening revealed the mother to be Group B Streptococcus positive while testing negative for Gonorrhea/Chlamydia, Hepatitis B Virus, HIV, Hepatitis C Virus, and VDRL, and was immune to Rubella. The maternal blood type was A positive. Additionally, the mother has a history of bipolar disorder, anxiety, and depression and was treated with fluoxetine during the pregnancy. There was cannabinoid use in early pregnancy, which was stopped upon learning of pregnancy. The diagnosis of Primrose syndrome was made prenatally based on fetal ultrasound showing absent septi pellucidi and non-visualized corpus callosum. Repeat fetal ultrasound confirmed the absence of the septi pellucidi, and a complete peri-callosal artery was unable to be demonstrated, raising concern for agenesis of the corpus callosum. This condition can be isolated, presenting little to no neurologic deficit, or it could be significant, thus warranting further investigation. Perinatal genetics consulted with the patient’s mother and recommended a fetal ultrasound (U/S) and MRI. Fetal ultrasounds revealed agenesis of the corpus callosum, associated colpocephaly, and mild unilateral right ventriculomegaly. The fetal MRI displayed complete agenesis of the corpus callosum, with lateral ventricles demonstrating a colpocephalic appearance and measuring 8-9 mm at the atrial level, bilaterally. The third and fourth ventricles were unremarkable, and there was no parenchymal signal alteration or intracranial hemorrhage. An amniocentesis sample, sent for fetal exome sequence analysis, tested positive for a heterozygous, c.1038 duplication (p.Ile347AspfsTer23), a likely pathogenic variant in ZBTB20 consistent with a diagnosis of Primrose syndrome. Parental testing did not identify the pathologic variant, classifying it as a de novo mutation that appeared spontaneously in the patient rather than being inherited from either parent. The fetal heart echo was normal, and alpha-fetoprotein was negative.

**Figure 1:**
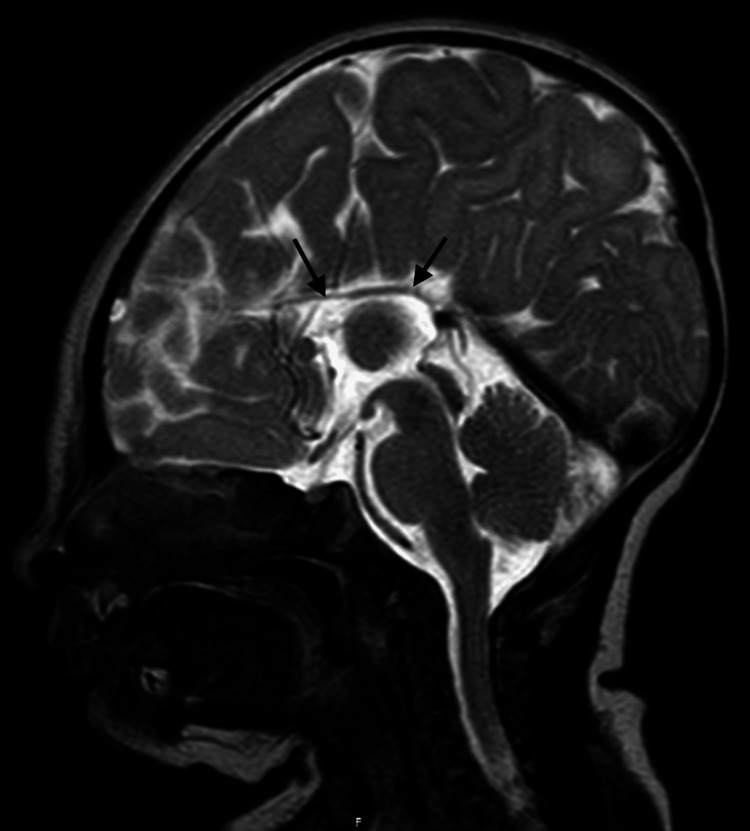
MRI of the brain, T2 sequence in the sagittal plane, demonstrating complete agenesis of the corpus callosum. The remaining brain parenchyma appears normal, with mild thinning of the white matter observed. Source: WellSpan York Hospital.

**Figure 2:**
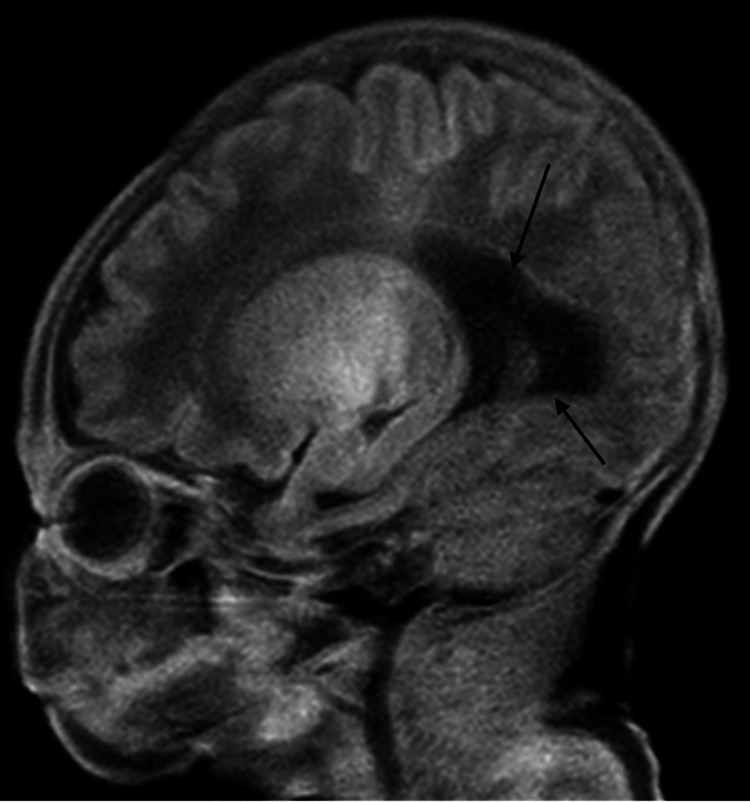
MRI of the brain, T1 sequence in the sagittal plane, illustrating colpocephaly. The image reveals prominence of the occipital horns of the lateral ventricles and mild thinning of the white matter, consistent with colpocephaly. Source: WellSpan York Hospital.

**Figure 3:**
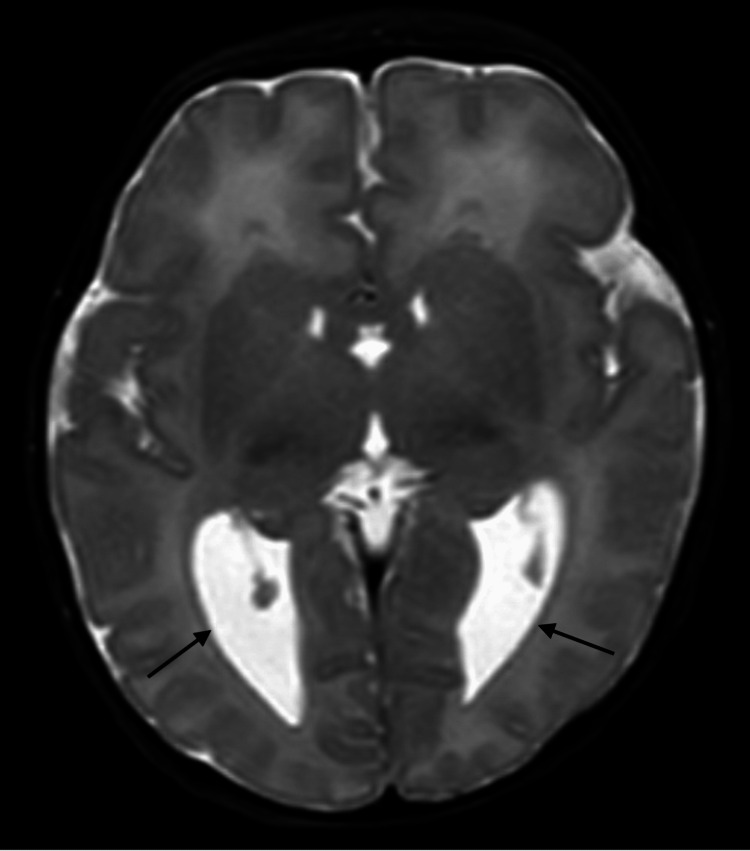
MRI of the brain, T2 sequence in the axial plane, displaying colpocephaly. Source: WellSpan York Hospital.

The newborn exam was notable for brachycephaly, slightly elevated hairline, slightly high-arched palate, and periorbital edema. Birth weight was 3270 g (53rd percentile), length was 52.5 cm (96th percentile), and head circumference was 36 cm (95th percentile). APGARS were 8 at 1 minute and 9 at 5 minutes. Pediatric neurology was consulted and saw the patient the following day. Regarding the neurologic exam, cranial nerve II demonstrated squint to bright light and tracking. Cranial nerves III, IV, and VI had positive doll's eye movements, with pupils constricting from 3 mm to 2 mm with light. Pupils were round and reactive bilaterally. Cranial nerve V showed a positive root reflex and opening of the mouth symmetrically. Cranial nerve VII showed symmetric facial grimace and orbicularis oculi strength 5 of 5 bilaterally. Cranial nerve VIII had positive doll's eye movements. Cranial nerves IX and X had a positive gag reflex, palate elevation in the midline, and good suck reflex. Cranial nerve XI had positive mild head lag. Cranial nerve XII had midline tongue thrust. Motor exam demonstrated good flexor tone with mild central hypotonia suggested by head lag. There was a movement of all extremities equally. The sensory exam had positive palmar and plantar grasp reflexes to light touch. Coordination showed a symmetric Moro reflex and an inability to hold the head parallel to the ground with ventral suspension. Stance and gait were non-ambulatory and age-appropriate. Biceps, triceps, patellar, and Achilles reflexes were 2+ and symmetric bilaterally, and toes were upgoing bilaterally. Critical congenital heart defect screening was negative. Newborn metabolic disease screening was unremarkable. The patient failed the initial newborn hearing screen but passed bilaterally prior to discharge. An MRI of the brain was performed at two days of life and showed agenesis of the corpus callosum (Figure 1) and mild colpocephaly (Figures 2 and 3). The patient had a follow-up at 13 days of life. The patient was feeding and gaining weight appropriately. She was initially breastfed at birth but switched to formula feeding prior to discharge and has since continued.

The patient was seen by pediatric neurology at two months of life. She continued to be feeding and growing well and had not experienced any seizures. She was noted to be cooing, tracking, and reacting to loud noises and had no excessive fisting of hands. The neurologic exam was significant for low muscle tone, head lag, and plagiocephaly, suggesting a preference for looking to the left. The patient did not have a consistent social smile. Weight was 5174 g (56th percentile), length was 55 cm (18th percentile), head circumference was 40.5 cm (97th percentile), and weight for length was in the 91st percentile. Neurology's plan included a referral to pediatric ophthalmology due to the risk of congenital cataracts and other intraocular problems, early intervention, physical therapy due to plagiocephaly, and a three-month follow-up with neurology. Additionally, if the patient began showing signs of hearing difficulties, such as not reacting to the conversation in the next three to four months, a referral to an ear, nose, and throat specialist was recommended to be made at that time to have hearing checked.

## Discussion

Since its discovery in 1982, Primrose syndrome has become both a recognizable and underdiagnosed condition, making awareness of its presentation crucial for early identification and proper management. It is a rare condition known to be caused by genetic changes in the ZBTB20 gene with characteristics of intellectual disability, autism spectrum disorder, craniofacial findings, corpus callosum anomalies, bilateral cataracts, hypothyroidism, diabetes mellitus, ectopic calcifications, hearing loss, and distal muscle wasting. Advances in molecular genetic testing have allowed for conditions such as Primrose syndrome to be identified [3]. Diagnosis of Primrose syndrome may be delayed because some clinical features, such as calcification of the external ears, cystic bone lesions, muscle wasting, and contractures, typically develop between 10 and 16 years of age [5]. Primrose syndrome follows an autosomal dominant inheritance pattern; most cases are sporadic. The association of ZBTB20 with Primrose syndrome was identified in a study of eight patients, all of whom were found to have heterozygous de novo missense variants [3]. This gene is located at chromosome position 3q13.31 and plays nonredundant roles in multiple organ systems [4]. It codes for a zinc finger protein-containing transcription factor involved in glucose metabolism, postnatal growth, and neurogenesis. ZBTB20 also plays an essential role in cognition, memory, and learning processes and has a transcription-repressive effect on numerous genes [3]. The genetic report for the patient stated that the c.1038dup variant in ZBTB20 seen in this case has not been reported in the literature or public variant repositories (ClinVar and LOVD) and is absent from population databases (gnomAD v2.1.1 and v3.1.2, TOPMed Freeze 8, All of Us). This suggests it is not a common benign variant in those populations. The c.1038dup variant in ZBTB20 is in exon 11 of this 12-exon gene. It is predicted to incorporate a premature stop codon, resulting in a protein lacking the entire C-terminal ZnF domain of the ZBTB20 protein [5]. Since Primrose syndrome is an autosomal dominant condition, genetic testing can identify whether the condition was inherited from a parent, as this may potentially impact future reproductive decisions. Genetic testing can also be done prenatally, as was done in this patient. Genetic counseling can help guide these decisions regarding family planning and potential early intervention. Diagnosis of Primrose syndrome is based on clinical and radiographic characteristic features and a heterozygous pathogenic variant in ZBTB20, as identified via molecular genetic testing. Various conditions may present similarly to Primrose syndrome, exhibiting developmental delay and multiple congenital abnormalities, such as Fragile X syndrome and DiGeorge syndrome. However, the genetic variant in ZBTB20, in conjunction with clinical and radiographic findings, allows Primrose syndrome to be distinguished from other conditions in the differential diagnosis. Clinical findings include characteristic craniofacial features such as brachycephaly, high anterior hairline, sparse eyebrows, deeply set eyes, down-slanted palpebral fissures, ptosis, high palate, and calcification of ear cartilage. Additionally, developmental delay, speech delay, intellectual disability, behavioral issues, macrocephaly at birth, hearing loss, cataracts, strabismus, cryptorchidism, and distal muscle atrophy are also characteristic clinical findings of Primrose syndrome [2]. Laboratory findings may include abnormal plasma acylcarnitine profile, urine organic acids, glucose metabolic profile, and increased serum alpha-fetoprotein levels. The patient, in this case, was noted to have normal plasma serum alpha-fetoprotein levels and glucose metabolic profile.

Imaging findings can include calcification of the external ear cartilage, as seen on a head CT, and cerebral calcification, primarily involving the basal ganglia. Brain MRI may show agenesis/dysgenesis of the corpus callosum, mild cerebral atrophy, and delayed myelination [2]. Radiographs may show unique skeletal manifestations, such as bitemporal bossing, slender bones with exaggerated metaphyseal flaring, mild epiphyseal dysplasia, and spondylar dysplasia. Management of Primrose syndrome is based on individual symptoms and presentation. Patients should be referred to neurology and ophthalmology for surveillance of ocular and neurologic deficits. Recommended assessment includes monitoring growth and development every six months and speech and developmental assessment every six months. Assessments for behavioral issues, seizures, and musculoskeletal complications should be conducted at each visit; brain stem evoked response audiometry should be performed annually, and annual evaluations should include fasting and postprandial blood glucose and hemoglobin A1c testing, alongside assessments for signs and symptoms of thyroid dysfunction [2]. Although longitudinal data are insufficient to determine life expectancy or outcomes, conditions such as pulmonary artery stenosis, testicular cancer, glaucoma, seizures, dysplastic hips, and distal muscle wasting have been noted [2]. The oldest reported individual is 53 years old [6].

## Conclusions

Primrose syndrome is a rare genetic condition with few reported occurrences. Our patient detailed in this case report demonstrates classic phenotypic and genotypic presentation of this syndrome, including a pathogenic variant in ZBTB20, absent corpus callosum on fetal ultrasound confirmed by MRI brain at birth, as well as high hairline, high-arched palate, and brachycephaly at birth. Diagnosis of Primrose syndrome is based on characteristic features and identification of heterozygous pathogenic variants in ZBTB20 via molecular genetic testing. Primrose syndrome management is supportive and based on individual symptoms and presentation. All infants should have involvement in pediatric neurology and annual evaluation for the development of associated medical conditions such as thyroid and glucose dysregulation. Data is insufficient to determine long-term outcomes or life expectancy.
